# Association between phase angle and coronary artery calcium score in patients on peritoneal dialysis

**DOI:** 10.3389/fnut.2022.912642

**Published:** 2022-08-04

**Authors:** Fabricio Moreira Reis, Maryanne Zilli Canedo da Silva, Nayrana Soares do Carmo Reis, Fabiana Lourenço Costa, Caroline Ferreira da Silva Mazeto Pupo da Silveira, Pasqual Barretti, Luis Cuadrado Martin, Silméia Garcia Zanati Bazan

**Affiliations:** Department of Internal Medicine, Botucatu Medical School-UNESP, São Paulo State University, Botucatu, Brazil

**Keywords:** bioelectrical impedance, phase angle, coronary artery calcium score, peritoneal dialysis, nutrition

## Abstract

**Introduction:**

The phase angle (PhA) has been used as a nutritional marker and predictor of mortality in patients on peritoneal dialysis (PD). The coronary artery calcium (CAC) score has shown to predict the incidence of acute myocardial infarction and death from cardiovascular disease in these patients. However, the association between PhA and CAC score in patients with PD is not well-established, which is the objective of this study.

**Materials and methods:**

Cross-sectional study with patients on PD, followed up at a University Hospital, between March 2018 and August 2019. PhA was evaluated by unifrequency bioimpedance (BIA). The CAC score was calculated based on cardiovascular computed tomography, considering positive when greater than or equal to 100 Agatston and negative when less than 100 Agatston.

**Results:**

We evaluated 44 patients on dialysis, with a mean age of 56 years and median time on dialysis therapy was 11.7 months. In the statistical analysis, a significant association was only observed between the CAC score and the PhA.

**Conclusion:**

The PhA is associated with a positive CAC score in patients with PD, and despite other factors, may be useful as a risk marker for coronary artery disease in this population.

## Introduction

It is known that 23–76% of patients on dialysis are malnourished, and 6–8% have severe malnutrition (1). Malnutrition is a risk factor for mortality (2, 3) and its causes are multifactorial, including hemodynamic, hormonal, inflammatory changes, and water overload, leading to protein energy wasting (PEW) (4).

Bioelectrical impedance (BIA) has been used to assess body composition (5–8). Its application is based on the electrical properties of the biological tissue (9, 10), evaluating their conductivity to an alternating electrical current, and has two components: resistance and reactance. The first determines the hydration status of the tissue (11–14) and the second represents the energy reserve of the cell membrane (which indirectly reflects the number of cells) (15). The angle formed by the vector sum of reactance and resistance is called the phase angle (PhA) (11–14), a composite marker influenced by hydration and integrity of the body cell membrane (16) that can be used as an index of nutritional assessment (15).

In patients with end-stage chronic kidney disease (CKD), coronary artery disease (CAD) is a major cause of morbidity and mortality. These patients are usually asymptomatic until the event of acute myocardial infarction (AMI) or sudden cardiac death (17). The evaluation of coronary calcification through coronary artery calcium (CAC) score, measured by computed tomography with multiple detectors, is a marker for atherosclerotic plaque burden and has shown to predict the incidence of AMI and death from cardiovascular disease (18).

In 2017, a study showed that PhA was a predictor of vascular calcification and arterial stiffness in patients on peritoneal dialysis (PD) (16). In 2021, a Chinese study that evaluated patients on hemodialysis found a relationship between PhA and coronary calcification (15). This relationship is not yet well-established, but it seems to involve the malnutrition-inflammation-atherosclerosis syndrome (13, 14, 19).

In this study, our objective was to evaluate the relationship between PhA and coronary calcification in patients with PD.

## Materials and methods

This cross-sectional study was approved by our Institutional Ethics and Research Committee (CAAE 80051517.1.0000.5411) and involved patients with CKD on PD of the Clinical Hospital of the Botucatu Medical School-UNESP, between March 2018 and August 2019.

Prevalent PD patients aged between 18 and 75 years, without previous coronary artery disease (CAD) or other overt atherosclerotic disease were included. Individuals with active or recent infections (< 7 days), autoimmune diseases, malignancy, or unstable heart disease (acute coronary syndrome, decompensated heart failure, and unstable arrhythmias) were not included in this study.

The registration of demographic and clinical data and the following complementary tests were performed with a maximum interval of 2 weeks: biochemical tests, nutritional assessment by bioimpedance (BIA), and anthropometry, calculation of dialysis adequacy (Kt/V), blood pressure measurement in upper extremities, ultrasound of carotid arteries, pulse wave velocity (PWV), ankle-brachial index (ABI), and CAC score.

### Nutritional assessment

Nutritional status was assessed by uni-and multifrequency bioimpedance (BIA). Unifrequency BIA was performed with a Biodynamic device (model 450) and multifrequency BIA with Fresenius Medical Care device–Body Composition Monitor (BCM) model.

In the unifrequency BIA, the values of PhA, total body water (TBW), intra and extracellular water, fat, and lean mass were considered. These values were determined by the device, and the formulas used to calculate total body water and intracellular water are based on those proposed by Kushner and Schoeller (20) and Cohn et al. (21). In the evaluation of the body composition monitor (BCM), the values of the hyperhydration index (overhydration-OH) were considered. This device measures the electrical response of 50 different types of frequencies from 5 to 1,000 kHz. An OH index > 1.1L were considered volume overload, according to Wizemann et al. (22).

The patient was instructed not to perform physical exercises within 24 h of the examination; to urinate, when with residual renal function, at least 30 min before the exam; not to drink alcohol in the 48 h before the test; and, during the examination, remain in the supine position. BIA measurements were performed with no dialysate in the peritoneal cavity, and with the patient in the supine position on a non-conductive surface.

### Coronary artery calcium score

The CAC score calculation was performed after a cardiovascular tomography scan (Multi slice, 64 channels, Optima, GE Medical Systems, Waukesha, WI, United States). Calcification consisted of a hyperattenuating lesion above the threshold of 130 Hounsfield units (HU) in an area of two or more adjacent pixels, observed in the coronary pathway. The product of the total area of calcium by a factor derived from the maximum attenuation (Maximal Computer Tomographic Number) is the calcium score published by Agatston et al. (23) and whose unit bears his name. The reported sensitivity and specificity in detecting this score are 98.7 and 100%, respectively (24). The images, including their quality and accuracy, were analyzed by a single examiner specializing in cardiovascular tomography, being “blinded” to the patient’s clinical, laboratory, and other complementary exam information. CAC score was considered positive when greater than or equal to 100 Agatston and negative when less than 100 Agatston.

### Statistical analysis

The sample size was calculated in 40 patients to detect a difference in the proportion of 30% between the groups, divided according to the median of the PhA, considering an error α of 5% and β of 20%.

Statistical analysis was performed using the SPSS version 23.0 (SPSS Inc., Chicago, IL, United States). Data were expressed as frequencies, mean ± *SD* or median and interquartile range, when appropriate. Statistical comparisons between the study groups (PhA ≤ 5.5° and > 5.5°) were performed using Student’s *t*-test for continuous variables and the chi-square test for categorical variables. Through a multivariate logistic regression model, associations were made between the study variables. The positive and negative predictive values, sensitivity, specificity, and accuracy, between the PhA and the CAC score were analyzed through the ROC curve (Receiver Operating Characteristic) and the calculated area under the ROC curve (AUC). The significance level adopted was *p* < 0.05.

## Results

**FIGURE 1:**
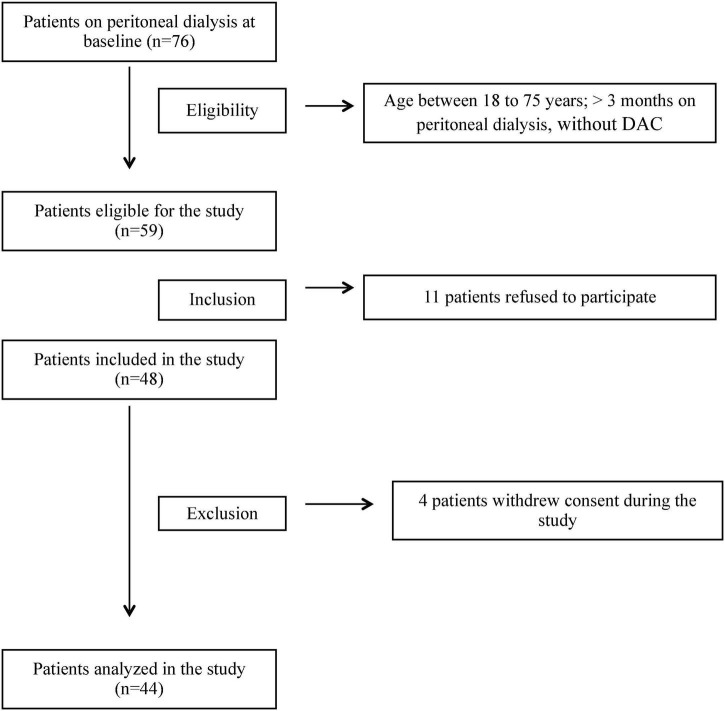
Flowchart of inclusion of patients in the study.

A total of 76 patients were on PD at the inclusion period, and 59 were eligible. Of these, 11 refused to participate. Therefore, 48 patients were included; however, four withdrew their consent in the middle of the study. Thus, 44 patients were analyzed (Figure 1).

The median age of patients was 56 years, most of them male, white, and with less than 9 years of schooling. Most patients were hypertensive and dyslipidemic. Patients with lower PhA (≤5.5°) were older (59, 56–68 years vs. 53, 38–62 years), had more diabetes (60 vs. 31%), and lower BMI (26.3 ± 3.3 kg/m^2^ vs. 28.7 ± 5.4 kg/m^2^), smoked more (13.3 vs. 6.9%) and were more sedentary (80 vs. 65.5%) when compared to the group with the highest PhA. However, none of these factors showed a statistically significant difference between the groups (Table 1).

**TABLE 1:** General characteristics of peritoneal dialysis (PD) patients.

	Total (*n* = 44)	Phase angle ≤5.5° (*n* = 15)	Phase angle >5.5° (*n* = 29)	*p*
Age (years)	56 (43–65)	59 (56–68)	53 (38–62)	0.393
Gender (% male)	54.5	60.0	51.7	0.752
Ethnic group (% white)	65.9	80.0	58.6	0.160
Diabetes (%)	40.9	60.0	31.0	0.105
Hypertension (%)	81.8	80.0	82.8	0.822
Dyslipidemia (%)	72.7	73.3	72.4	0.948
Body mass index (kg/m^2^)	27.5 ± 4.8	26.3 ± 3.3	28.7 ± 5.4	0.234
Family story DAC (%)	22.9	20.0	24.0	0.799
Smoking (%)	9.1	13.3	6.9	0.481
Alcohol consumption (%)	9.1	13.3	6.9	0.481
Physical level (% sedentary)	70.5	80.0	65.5	0.550
Dialysis vintage (months)	11.7 (6.7–23.9)	11.3 (6.9–25.8)	12.6 (6.6–26.2)	0.525
Underlying condition				
Diabetes (%)	27.3	26.7	27.6	0.597
Hypertension (%)	31.8	33.3	31.0	
Glomerulopathies (%)	20.5	13.3	24.1	
Others (%)	20.4	26.7	17.3	
Previous hemodialysis (%)	13.6	13.3	13.8	0.966
Total Kt/V	2.2 (1.7–2.5)	2.8 (1.8–2.5)	2.2 (1.8–2.5)	0.988
Urine output (ml)	1071 ± 686	827 ± 535	1207 ± 732	0.086
Glomerular filtration rate (ml/min/1.73 m^2^)	5.5 (2.1–8.0)	3.2 (1.9–7.4)	5.6 (2.8–8.7)	0.525

Data expressed as mean ± standard deviation or median (interquartile range). Student’s t-test or chi-square.

DAC, coronary artery disease; Kt/V, dialysis adequacy.

Almost a third (31.8%) of patients had hypertension as the underlying disease for CKD, followed by diabetes (27.3%). The median dialysis vintage was 11.7 (6.7–23.9) months. The groups PhA ≤ 5.5° and > 5.5° were similar in terms of these variables, except for the urinary output that was higher in the group with PhA > 5.5 (1,207 vs. 827 ml), almost reaching statistical significance (Table 1).

In the evaluation of unifrequency BIA and OH parameters, patients with PhA ≤ 5.5° presented higher percentages of extracellular water and OH index and a lower percentage of intracellular water. The percentages of lean mass, fat mass, and total body water were similar between the groups (Table 2).

**TABLE 2:** Bioimpedance parameters of peritoneal dialysis (PD) patients.

	Total (*n* = 44)	Phase angle ≤5.5° (*n* = 15)	Phase angle >5.5° (*n* = 29)	*p*
Lean mass (%)	70.0 ± 7.2	69.3 ± 7.2	70.4 ± 7.3	0.624
Fat mass (%)	30.0 ± 7.2	30.7 ± 7.2	29.6 ± 7.3	0.624
Intracellular water (%)	53.2 ± 3.6	50.1 ± 2.7	54.8 ± 3.0	<0.001
Extracellular water (%)	46.8 ± 3.6	49.9 ± 2.7	45.2 ± 3.0	<0.001
Total body water (litres)	37.3 ± 8.1	34.6 ± 6.8	38.7 ± 8.5	0.112
Overhydration (litres)	0.5 ± 1.5	1.5 ± 1.5	0.0 ± 1.2	0.001

Data expressed as mean ± standard deviation. Student’s t-test or chi-square.

When analyzing the laboratory tests, the groups PhA ≤ 5.5° and > 5.5° were similar (Table 3). As for the atherosclerosis markers, only the percentage of positive CAC score (73.3 vs. 20.7%) showed a significant difference between the PhA ≤ 5.5° and > 5.5° groups (Table 4).

**TABLE 3:** Laboratory tests of peritoneal dialysis (PD) patients.

	Total (*n* = 44)	Phase angle ≤5.5° (*n* = 15)	Phase angle > 5.5° (*n* = 29)	*p*
Hemoglobin (g/dL)	11.7 ± 1.1	11.9 ± 1.1	11.5 ± 1.1	0.249
Urea (mg/dL)	103.6 ± 24.9	106.6 ± 26.2	102.2 ± 21.4	0.550
Creatinine (mg/dL)	9.3 ± 3.1	8.3 ± 2.2	9.3 ± 3.3	0.285
Corrected calcium (mg/dL)	9.2 ± 0.7	8.9 ± 0.8	9.3 ± 0.7	0.078
Magnesium (mmol/L)	2.0 ± 0.3	2.0 ± 0.3	2.0 ± 0.3	0.749
Phosphorus (mg/dL)	5.3 ± 1.1	5.1 ± 1.2	5.5 ± 1.2	0.243
Calcium × Phosphorus (mg^2^/dL^2^)	48.9 ± 10.5	44.7 ± 9.9	51.1 ± 10.3	0.057
HDL (mg/dL)	36.5 (30.7–43.2)	33 (30–38)	36 (32–53)	1.000
LDL (mg/dL)	73.7 ± 29.3	85.2 ± 30.0	77.0 ± 41.7	0.504
Triglycerides (mg/dL)	153.5 (117.0–210.7)	208 (118–240)	159 (124–287)	0.525
Total cholesterol (mg/dL)	147.7 ± 34.6	160.4 ± 34.1	154.7 ± 49.9	0.693
Glycosylated hemoglobin (%)	5.4 (5.1–6.3)	6.2 (5.5–6.6)	5.2 (5.0–5.9)	0.828
Albumin (g/dL)	3.7 ± 0.4	3.6 ± 0.5	3.7 ± 0.3	0.438
Uric acid (mg/dL)	6.0 ± 1.3	5.7 ± 1.2	6.4 ± 1.0	0.115
Alkaline phosphatase (U/L)	76.0 (68.7–127.2)	76 (67–143)	72 (47–96)	0.525
PTH (mg/dL)	241.0 (164.5–356.0)	311 (145–377)	201 (171–371)	0.326
Vitamin D (ng/mL)	25.8 (20.0–31.1)	25.7 (21.8–31.9)	25.3 (16.4–29.3)	0.897
High-sensitivity troponin (ng/L)	7.5 (2.3–17.1)	11.5 (7.2–16.2)	2.9 (1.8–16.6)	0.056
NT-Pro-BNP (pg/mL)	291 (205–411)	299 (146–539)	283 (182–678)	1.000
Interleukin 6 (pg/mL)	14.6 (8.5–27.9)	13.9 (5.3–16.2)	25.8 (9.6–37.7)	0.659
TNF-α (pg/mL)	4.6 (0.6–13.6)	3.5 (0.0–14.6)	8.5 (4.9–18.9)	0.970
Ultrasensitive CRP (mg/L)	1.9 (0.5–5.1)	3.4 (0.9–9.1)	1.2 (0.0–4.9)	0.743

Data expressed as mean ± standard deviation or median (interquartile range). Student’s t-test or Mann-Whitney.

HDL, high-density lipoprotein; LDL, low-density lipoprotein; NT-Pro-BNP, N-terminal prohormone B-type natriuretic peptide; PTH, parathyroid hormone; CRP, c-reactive protein; TNFα, tumor necrosis factor-alpha.

**TABLE 4:** Atherosclerosis markers of peritoneal dialysis (PD) patients.

	Total (*n* = 44)	Phase angle ≤5.5° (*n* = 15)	Phase angle >5.5° (*n* = 29)	*p*
CAC score positive	38.6	73.3	20.7	0.001
VOP femoral (m/s)^a^	10.5 ± 4.0	11.1 ± 4.4	9.2 ± 3.5	0.166
Brachial ankle index	1.0 ± 0.2	1.1 ± 0.2	1.0 ± 0.1	0.573
Difference PAS-MMSS	15.9	20.0	13.8	0.594
EMIC left (cm)^b^	0.7 (0.6–0.8)	0.7 (0.6–1.0)	0.7 (0.6–0.7)	0.344
EMIC right (cm)^b^	0.7 ± 0.1	0.7 ± 0.2	0.7 ± 0.1	0.106
Carotid plaque left^b^	36.1	54.5	28.0	0.127
Carotid plaque right^b^	44.4	63.6	36.0	0.124

Data expressed as mean ± standard deviation or median (interquartile range). Student’s t-test or chi-square.

^a^n total = 36; n Pha > 5.5° = 23; n PhA ≤ 5.5° = 13.

^b^n total = 36; n PhA > 5.5° = 25; n PhA ≤ 5.5° = 11.

CAC, coronary artery calcium; EMIC, average carotid intimal thickness; PAS, systolic blood pressure; VOP, pulse wave speed.

As some variables are known to be associated with PhA, such as age, sex, diabetes, glomerular filtration rate, BMI, hemoglobin, physical activity, calcium x phosphorus, PTH, and ultrasensitive CRP a hierarchical multiple logistic regression was performed to predict positive CAC score. In this evaluation, only PhA remained an independent predictor for positive CAC scores (Table 5).

**TABLE 5:** Hierarchical multiple logistic regression with some variables related to the phase angle to predict positive ECAC.

	Variables	OR	*p*	Confidence interval	
1° step	Age (years)	1.168	0.315	0.862	1.583
	Gender (% male)	2.087	0.689	0.057	76.780
	Diabetes (%)	0.011	0.219	0.000	14.921
	Glomerular filtration rate (ml/min/1,73 m^2^)	1.204	0.397	0.784	1.848
	Body mass index (kg/m^2^)	0.708	0.470	0.278	1.806
	Hemoglobin (g/dL)	0.672	0.695	0.093	4.877
	Physical level (% sedentary)	2.888	0.835	0.000	62705.095
	Calcium × Phosphorus (mg^2^/dL^2^)	1.151	0.331	0.866	1.529
	PTH (mg/dL)	1.008	0.286	0.993	1.023
	Ultrasensitive CRP (mg/L)	2.079	0.940	0.882	4.900
	Phase angle (°)	0.007	0.013	0.000	1.510
7° step	PTH (mg/dL)	1.010	0.060	1.000	1.020
	Ultrasensitive CRP (mg/L)	1.502	0.076	0.959	2.353
	Age (years)	1.096	0.102	0.982	1.224
	Diabetes (%)	0.078	0.081	0.004	1.367
	Phase angle (°)	0.055	0.008	0.007	0.462

Hierarchical multiple logistic regression. Model: Age, Gender, DM, Glomerular filtration rate, BMI, Hemoglobin, Physical level, Calcium × Phosphorus, PTH, Ultrasensitive CRP, and Phase angle.

DM, diabetes; BMI, body mass index; OR, odds ratio.

**FIGURE 2:**
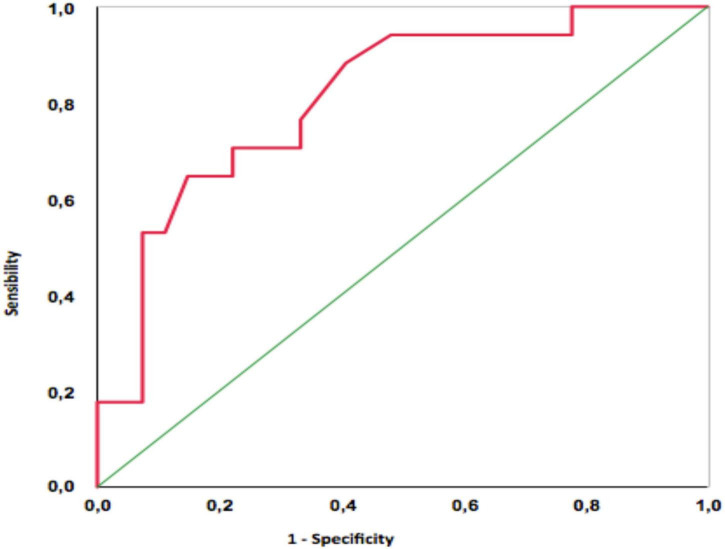
ROC curve for diagnosis of positive CAC score from the phase angle information.

In the analysis of the ROC curve for the diagnosis of a positive CAC score from the PhA, the area under the curve was.81 (CI: 0.68–0.94; *p* < 0.01). The best cut-off point was with PhA ≤ 5.5°, which showed a sensitivity of 64.7% and a specificity of 85.2%. Also, when the PhA was ≤ 7.2°, the sensitivity was 100% (Figure 2).

## Discussion

This study showed that there is an inverse correlation between the PhA and CAC score in patients on PD, even after adjusting for several variables such as age, sex, diabetes, glomerular filtration rate, BMI, hemoglobin, physical activity, calcium x phosphorus, PTH, and ultrasensitive CRP.

The best cut-off point was with a PhA ≤ 5.5°, which had a sensitivity of 64.7% and a specificity of 85.2% to predict a positive CAC score. Also, when the PhA was ≤ 7.2°, the sensitivity was 100%.

A recent study (15) found the same result in hemodialysis patients. Sarmento-Dias et al. (16) studying patients with PD showed that the PhA predicts arterial stiffness and vascular calcification. However, we did not find any study in the literature relating the phase angle with coronary calcification in PD patients.

One of the hypotheses for the relationship between PhA and CAC score is that the first reflects the nutritional status (25) and malnutrition plays an important role in the development of cardiovascular diseases, due to the malnutrition-inflammation-atherosclerosis syndrome (26). Saitoh et al. (27) showed a correlation positive between the PhA with the percentage of lean mass and BMI, and negative with protein-energy malnutrition. Leal-Alegre et al. (28) found 29% of PEW in patients with vascular calcification undergoing PD. In 2021 (15), a study showed that the nutritional status of hemodialysis patients with vascular calcification was worse than those without calcification. In our study, there was no relationship between inflammatory markers (CRP, interleukins, and TNF-alpha) and PhA. However, some authors report that malnutrition may be a risk factor for cardiovascular mortality, independent of inflammation (29), and may have other still unknown mechanisms involved.

Another hypothesis is that the PhA is influenced by the hydration state (16), and the smaller the PhA, the higher the level of extracellular fluid. Excess extracellular fluid (ECF) results in pathological mechanical stimuli in vascular endothelium and smooth muscle cells. Such stimuli release angiotensin II, increase superoxide production, and reduce nitric oxide bioavailability, leading to atherosclerosis, and vascular calcification (30). In this study, higher levels of ECF and OH index showed a direct relationship with lower PhA and positive CAC scores.

The strengths of this study are the multivariate and hierarchical regression analysis for variables that could influence the CAC score in the population studied, and a large number of variables analyzed, enabling a better understanding of the relationship studied. Limitations include the cross-sectional study design, which does not allow establishing a causal relationship between the PhA and CAC score variables. Also, the small sample size, even though just peritoneal dialysis patients without overt atherosclerotic disease were included, and the fact that it was performed in a single center.

## Data availability statement

The raw data supporting the conclusions of this article will be made available by the authors, without undue reservation.

## Ethics statement

The studies involving human participants were reviewed and approved by the research Ethics Committee of Botucatu Medical School. The patients/participants provided their written informed consent to participate in this study.

## Author contributions

FR was responsible for the research idea and study design. FR, FC, and MS performed data acquisition. FR, MS, NR, LM, FC, and CS performed data analysis and interpretation, involved in statistical analysis, and drafted the manuscript. PB, LM, and SB were responsible for supervision and mentorship. All authors provided intellectual content to the work and gave final approval of the version to be published.
